# Acute Necrotizing Pancreatitis Presenting With Extensive Unilateral Lower Limb Myositis, Fasciitis, and Sterile Reactive Knee Synovitis: A Rare Extra-pancreatic Manifestation

**DOI:** 10.7759/cureus.114185

**Published:** 2026-08-08

**Authors:** Vamsi K Uppati, Anil K Abburi, Lava K Pydi

**Affiliations:** 1 Gastroenterology, Great Eastern Medical School and Hospital, Srikakulam, IND; 2 Radiology, Great Eastern Medical School and Hospital, Srikakulam, IND; 3 Internal Medicine, Great Eastern Medical School and Hospital, Srikakulam, IND

**Keywords:** acute necrotizing pancreatitis, fasciitis, myositis, pancreatic panniculitis, reactive synovitis

## Abstract

Musculoskeletal manifestations of acute pancreatitis are uncommon and are most frequently described as pancreatic panniculitis or pancreatic panniculitis-polyarthritis (PPP) syndrome. Diffuse inflammatory myositis with fascial involvement is exceedingly rare and may mimic cellulitis, pyomyositis, septic arthritis, necrotizing fasciitis, or deep venous thrombosis, leading to diagnostic uncertainty and potentially unnecessary surgical intervention.

A 44-year-old man presented with severe epigastric pain radiating to the back for 15 days, followed by fever and rapidly progressive painful swelling of the left thigh, knee, and leg. Serum lipase was elevated (867 U/L). Contrast-enhanced computed tomography (CECT) demonstrated acute necrotizing pancreatitis with <30% pancreatic necrosis, multiple acute necrotic collections, a modified CT severity index (MCTSI) of 8/10, and partial thrombosis of the splenic, superior mesenteric, and portal veins. Venous Doppler excluded deep venous thrombosis. Magnetic resonance imaging (MRI) of the thigh and knee revealed diffuse multicompartment inflammatory myositis, extensive fascial fluid tracking, diffuse subcutaneous panniculitis, and mild knee joint effusion without osteomyelitis or drainable abscess. Knee ultrasonography showed quadriceps edema with mild suprapatellar effusion. Synovial fluid analysis demonstrated a leukocyte count of only 250 cells/mm³, while blood and urine cultures remained sterile. Based on the clinicoradiological findings and exclusion of infection, a diagnosis of pancreatic enzyme-mediated lower limb myositis, fasciitis, panniculitis, and sterile reactive synovitis was made. The patient was treated conservatively with supportive care for acute pancreatitis, therapeutic anticoagulation for splanchnic venous thrombosis, analgesia, nutritional support, and physiotherapy, resulting in progressive clinical recovery without surgical intervention.

This case expands the recognized musculoskeletal spectrum of acute pancreatitis by demonstrating extensive MRI-confirmed myositis, fasciitis, panniculitis, and sterile synovitis occurring concurrently with necrotizing pancreatitis and splanchnic venous thrombosis. Recognition of this rare entity is essential because it closely mimics soft tissue infection but responds to treatment directed at the underlying pancreatic disease. Multimodality imaging and careful exclusion of infective etiologies are critical to establishing the diagnosis and avoiding unnecessary operative management.

## Introduction

Acute pancreatitis is one of the most common gastrointestinal emergencies, with an annual incidence ranging from 13 to 45 cases per 100,000 population worldwide [1]. Although most episodes are mild and self-limiting, approximately 20% of patients develop moderately severe or severe acute pancreatitis, characterized by persistent organ failure and local or systemic complications [1-3]. The Revised Atlanta Classification provides a standardized framework for disease severity and defines local complications, such as acute necrotic collections and walled-off necrosis [2], which are associated with increased morbidity and mortality.

The systemic inflammatory response in acute pancreatitis extends beyond the pancreas and can affect multiple organ systems. Pulmonary complications, acute kidney injury, cardiovascular dysfunction, and splanchnic venous thrombosis are well-recognized manifestations of severe disease [3]. In contrast, musculoskeletal complications are distinctly uncommon and therefore often remain unrecognized during the initial evaluation [4].

Pancreatic panniculitis is the best-described musculoskeletal and dermatological manifestation of pancreatic disease [4], occurring in only 0.3%-3% of affected patients [5]. It is characterized by painful erythematous subcutaneous nodules resulting from enzymatic fat necrosis and is most commonly associated with pancreatic acinar cell carcinoma, although it has also been reported in acute pancreatitis, chronic pancreatitis, pancreatic trauma, and pancreatic pseudocysts. Histopathologically, pancreatic panniculitis demonstrates lobular fat necrosis with characteristic anucleate “ghost” adipocytes, reflecting the lipolytic effects of circulating pancreatic enzymes [5].

An even rarer manifestation is pancreatic panniculitis-polyarthritis (PPP) syndrome, a clinicopathological entity characterized by the triad of pancreatic disease, panniculitis, and inflammatory arthritis [4]. Fewer than 100 cases have been described in the literature, and musculoskeletal manifestations frequently precede abdominal symptoms [4], resulting in delayed diagnosis and unnecessary orthopedic or surgical interventions.

Diffuse inflammatory myositis and fasciitis associated with acute pancreatitis are exceptionally rare [6]. Only isolated case reports have documented magnetic resonance imaging (MRI)-confirmed skeletal muscle involvement, and the underlying pathogenesis remains incompletely understood. Current evidence suggests that pancreatic enzymes - particularly lipase and phospholipase A₂ - gain access to the systemic circulation through increased vascular permeability during severe pancreatitis. These enzymes hydrolyze triglycerides within adipose tissue, generating free fatty acids that induce fat necrosis, endothelial injury, cytokine release, and secondary inflammation involving subcutaneous tissue, fascia, periarticular structures, and skeletal muscle [5,6].

The clinical presentation of pancreatic enzyme-mediated myositis is particularly challenging because patients often present with painful unilateral limb swelling, erythema, fever, and restricted joint movement, closely resembling cellulitis, pyomyositis, septic arthritis, necrotizing fasciitis, deep venous thrombosis, or compartment syndrome. Consequently, many patients receive prolonged antibiotic therapy or undergo unnecessary surgical exploration before the pancreatic origin of the disease is recognized.

MRI plays a pivotal role in differentiating inflammatory myositis from infective soft tissue disorders. Typical findings include diffuse T2-weighted muscle hyperintensity, fascial edema, subcutaneous inflammatory changes, and post-contrast enhancement without a well-defined drainable abscess. When interpreted in conjunction with cross-sectional abdominal imaging, microbiological investigations, and clinical findings, MRI can facilitate early diagnosis and prevent avoidable operative intervention.

Because pancreatic enzyme-mediated musculoskeletal manifestations closely mimic cellulitis, pyomyositis, septic arthritis, necrotizing fasciitis, and deep venous thrombosis, patients may receive unnecessary antibiotics or surgical intervention before the correct diagnosis is established. Only a limited number of cases describing pancreatic enzyme-mediated myositis and fasciitis have been reported, highlighting the rarity of this presentation.

We report a rare case of acute necrotizing pancreatitis presenting with extensive unilateral lower limb inflammatory myositis, fasciitis, panniculitis, and sterile reactive knee synovitis. The objective of this report is to increase awareness of this uncommon extra-pancreatic manifestation and emphasize the importance of early recognition to avoid unnecessary invasive interventions.

## Case presentation

A 44-year-old man with no previous history of pancreatic disease presented with severe epigastric pain radiating to the back for 15 days. The pain was continuous, associated with multiple episodes of non-bilious vomiting, and progressively worsened. Three days before presentation, he developed high-grade fever followed by rapidly progressive painful swelling of the left lower limb involving the thigh, knee, leg, and foot, resulting in difficulty bearing weight and restricted movement. There was no history of preceding trauma, intramuscular injection, strenuous exercise, insect bite, or similar previous episodes.

The pancreatitis was alcohol induced. The patient was immunocompetent with no history of HIV infection, malignancy, organ transplantation, or immunosuppressive therapy.

On examination, the patient appeared ill. Abdominal examination revealed epigastric tenderness without guarding or rebound tenderness. Examination of the left lower limb demonstrated diffuse unilateral swelling extending from the proximal thigh to below the knee with tense edema, warmth, marked tenderness, and painful restriction of knee movements. There were no bullae, skin necrosis, crepitus, discharging sinuses, or neurological deficits. Distal arterial pulses were palpable with normal capillary refill (Figure 1).

**Figure 1:**
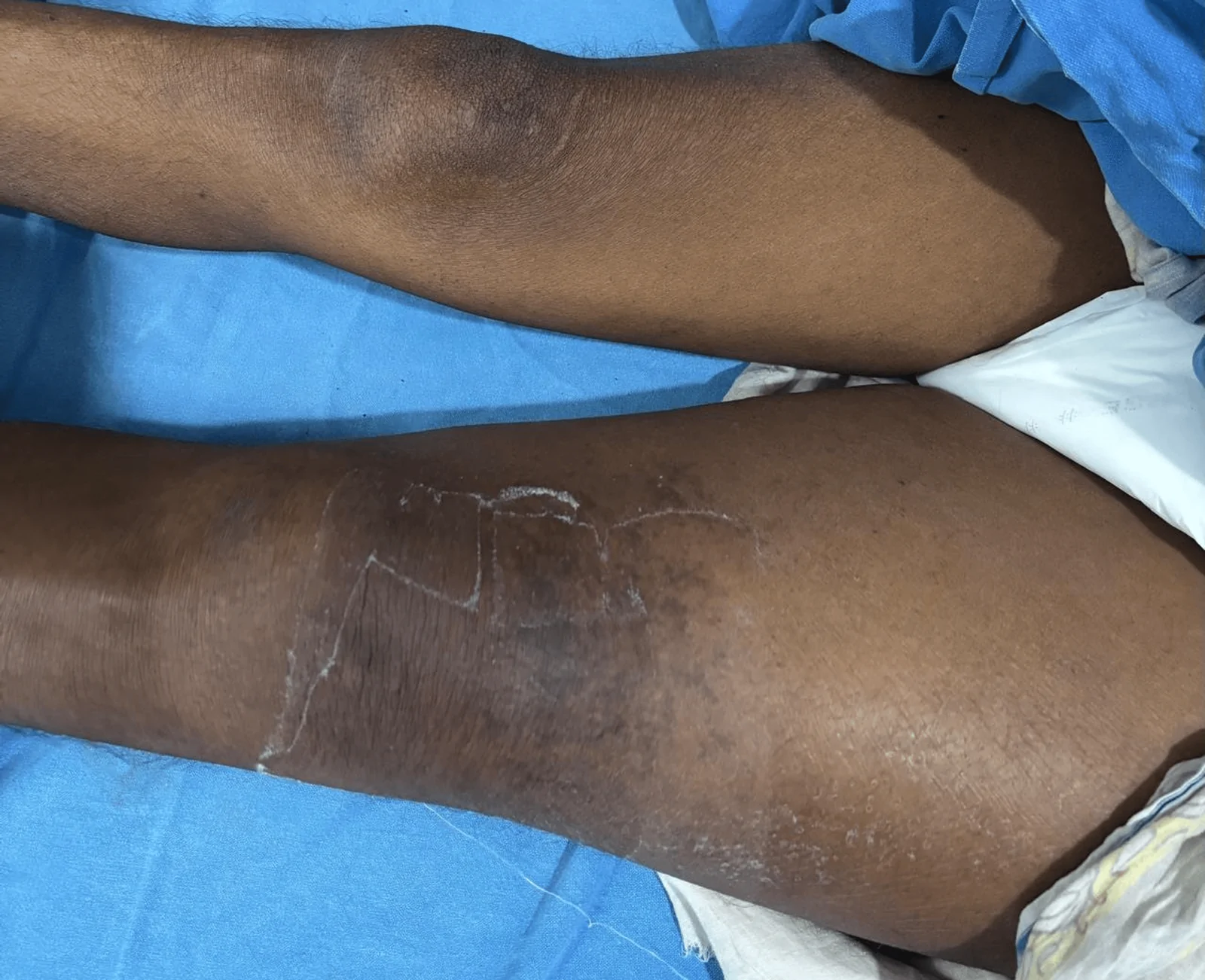
Clinical presentation of the left lower limb. Clinical image shows diffuse unilateral swelling of the left lower limb extending from the proximal thigh to below the knee with tense edema and overlying skin changes.

Initial laboratory evaluation revealed a serum lipase level of 867 U/L, confirming acute pancreatitis. Because of the disproportionate unilateral limb swelling, differential diagnoses included deep venous thrombosis, cellulitis, pyomyositis, septic arthritis, and necrotizing fasciitis.

Contrast-enhanced computed tomography (CECT) of the abdomen demonstrated acute necrotizing pancreatitis with less than 30% pancreatic necrosis, multiple acute necrotic collections involving the lesser sac, gastrohepatic ligament, and gastrosplenic ligament, and along the gastroduodenal artery, with the largest collection measuring approximately 6.5 × 5.8 cm. The modified CT severity index (MCTSI) was 8/10. Partial thrombosis of the portal vein, splenic vein, and superior mesenteric vein was also identified without evidence of pseudoaneurysm or active hemorrhage (Figure 2).

**Figure 2:**
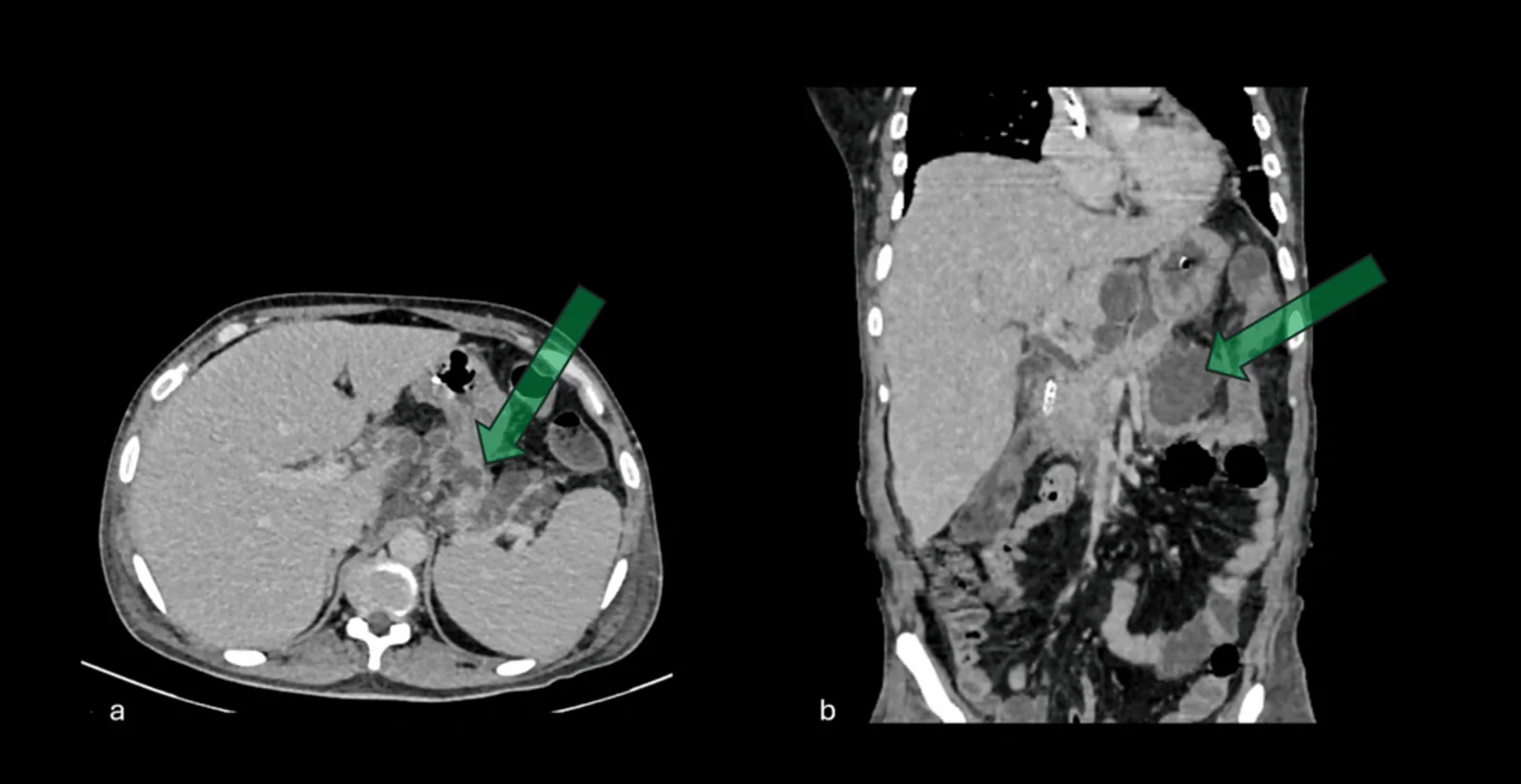
Contrast-enhanced CT of the abdomen demonstrating acute necrotizing pancreatitis. Axial (a) and coronal (b) CECT images show multiple acute necrotic collections (green arrows) involving the pancreas and the surrounding peripancreatic regions. CECT: contrast-enhanced computed tomography

Duplex Doppler ultrasonography of the left lower limb demonstrated normal arterial flow and excluded deep venous thrombosis. Diffuse subcutaneous edema extending from the upper thigh to the dorsum of the foot was noted (Figure 3). 

**Figure 3:**
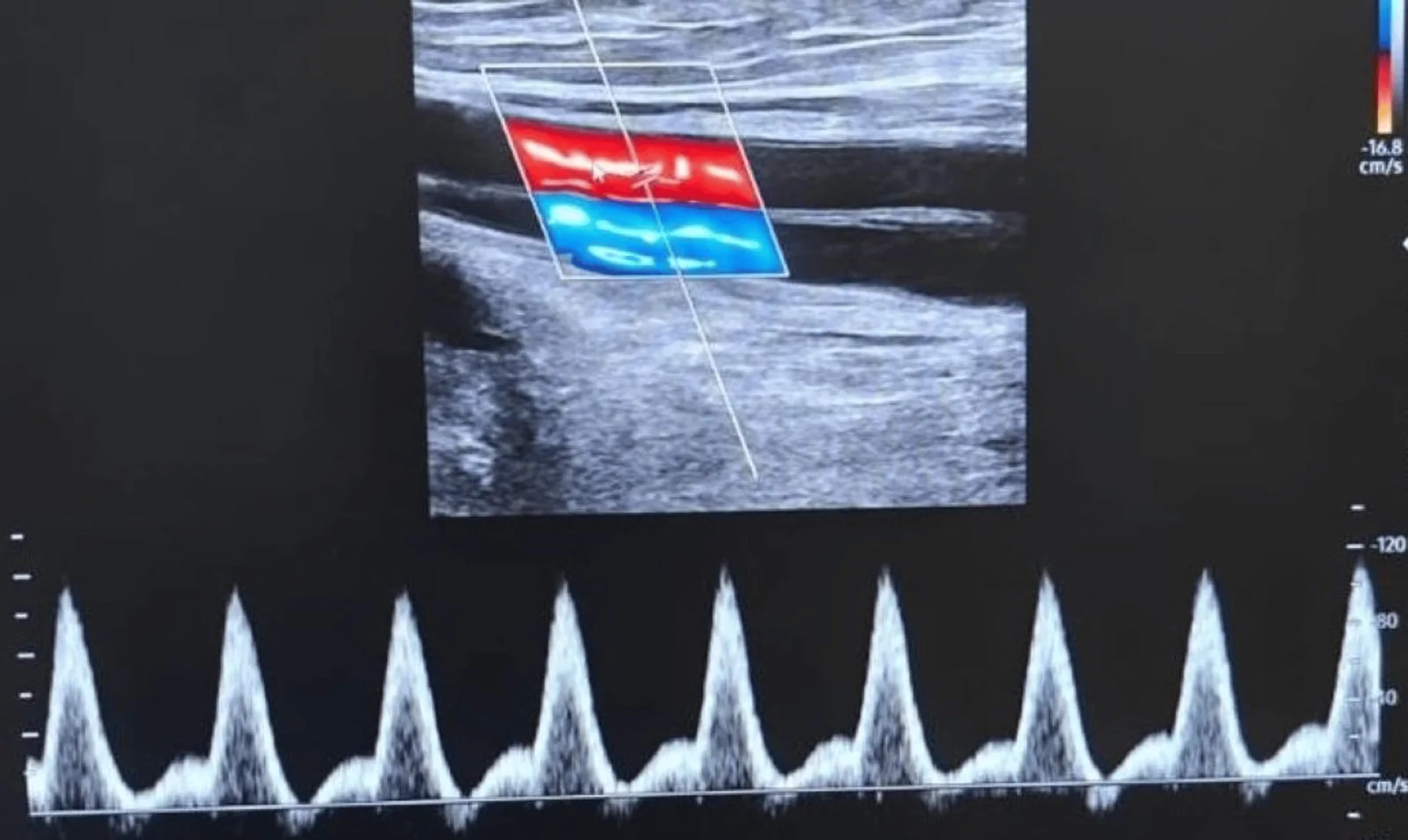
Duplex Doppler ultrasonography of left lower limb. Duplex Doppler ultrasonography demonstrating preserved arterial and venous flow without evidence of deep venous thrombosis.

Ultrasonography of the knee demonstrated bulky hyperechoic quadriceps musculature consistent with inflammatory edema, mild suprapatellar joint effusion, and synovial thickening without features of septic arthritis.

Given the extensive soft tissue involvement, contrast-enhanced MRI of the left thigh and knee was performed. MRI demonstrated diffuse multicompartment inflammatory myositis involving the anterior, medial, and posterior compartments of the thigh (Figure 4a-4c).

**Figure 4:**
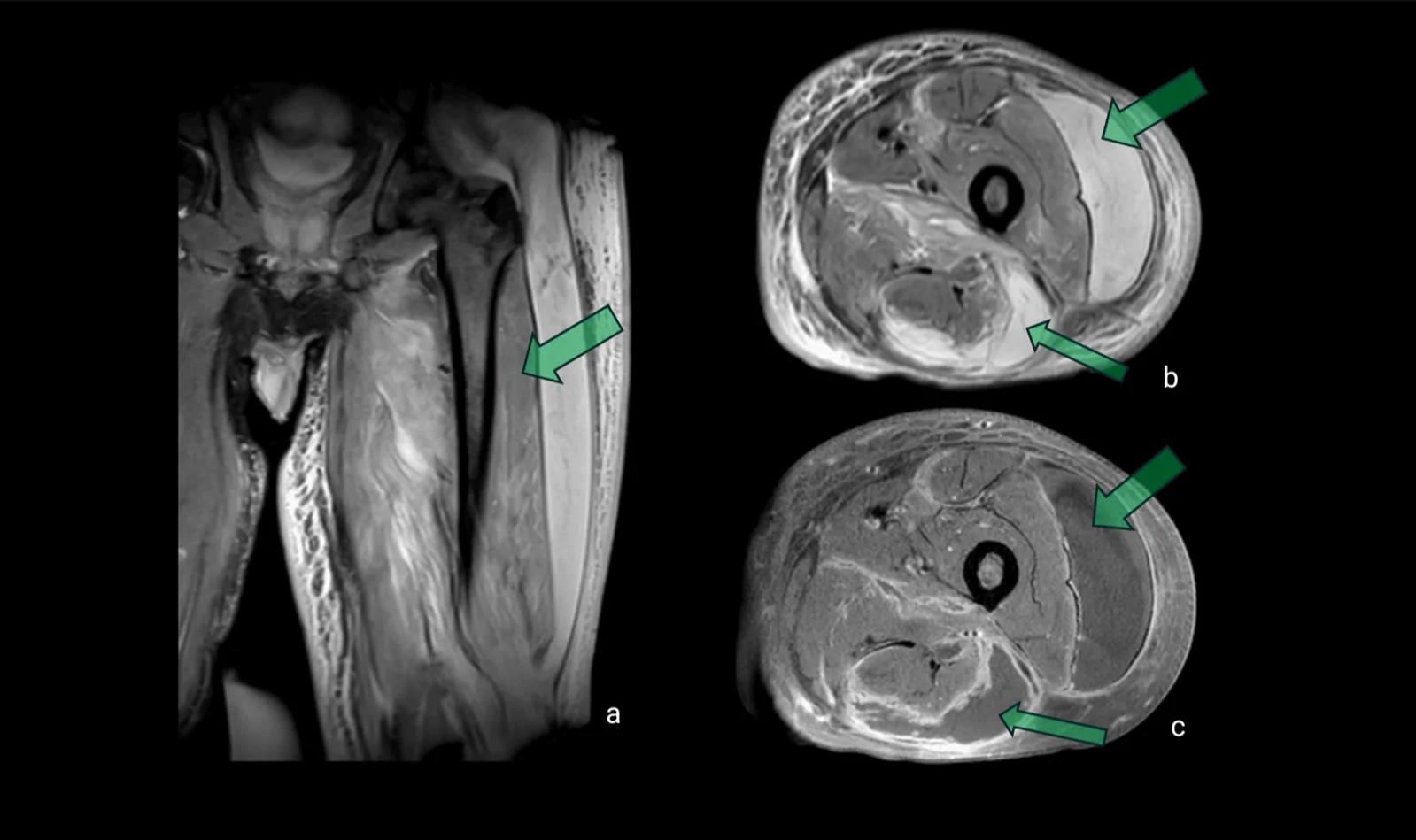
Contrast-enhanced MRI of the left thigh. Coronal (a) and axial (b) PDFS sections, and post-contrast axial T1 (c) show hyperintense collections (green arrows) beneath the fascia tracking along the thigh in the anterior and posterior compartments with edema involving the muscles of the thigh. PDFS: proton density fat-saturated

MRI of the left lower limb using proton density fat-saturated (PDFS) sequences demonstrated extensive fascial fluid tracking, diffuse subcutaneous inflammatory changes suggestive of panniculitis, and mild knee joint effusion without evidence of osteomyelitis or a drainable abscess (Figure 5).

**Figure 5:**
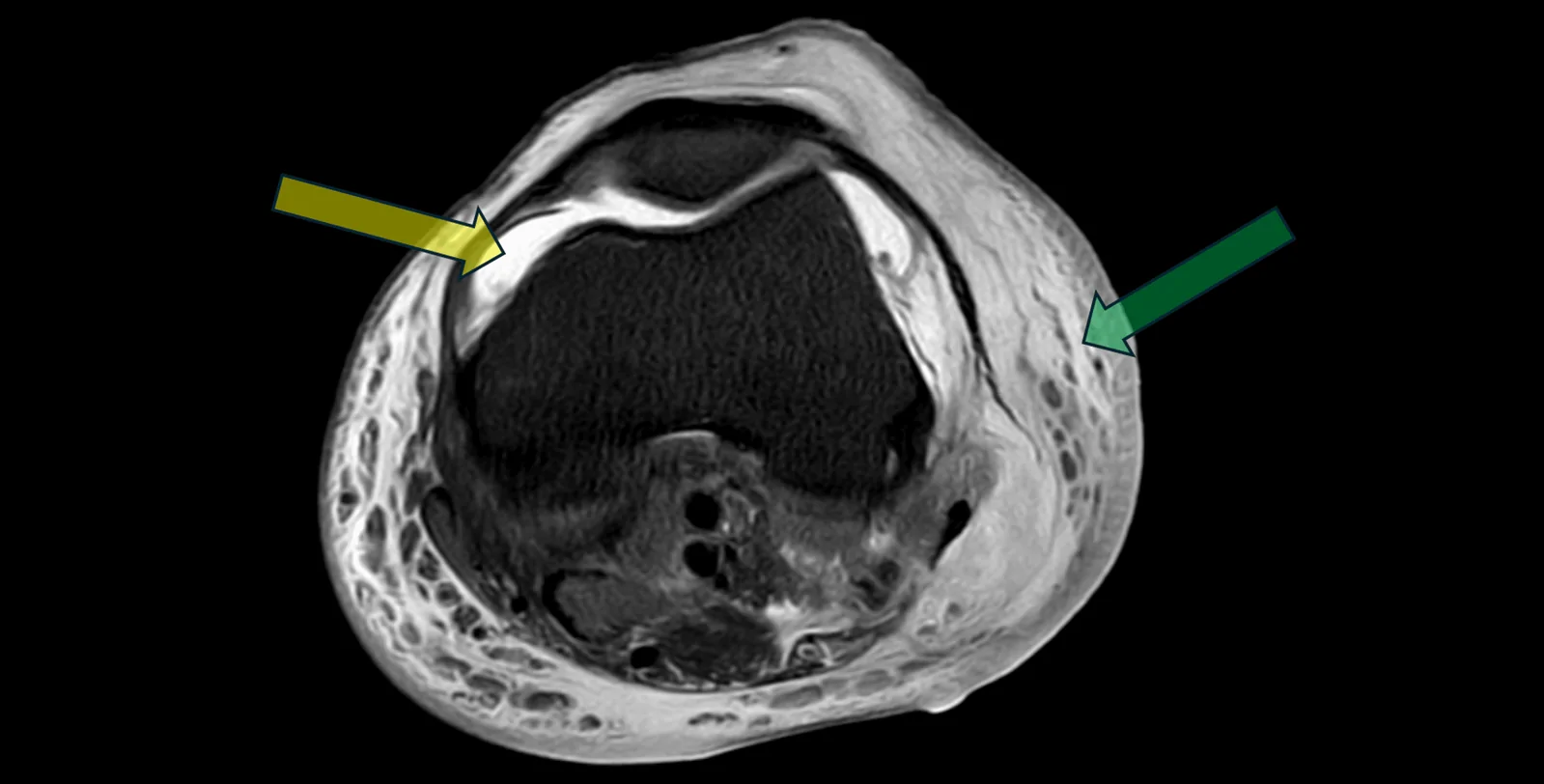
Contrast-enhanced MRI of the left knee. Axial PDFS sequence shows diffuse subcutaneous edema (green arrow) and mild joint effusion (yellow arrow). PDFS: proton density fat-saturated

Diagnostic aspiration of the knee yielded inflammatory synovial fluid with a leukocyte count of 250 cells/mm³ (80% neutrophils and 20% lymphocytes). Blood and urine cultures remained sterile throughout hospitalization. Based on the clinicoradiological findings, negative microbiological evaluation, and exclusion of deep venous thrombosis and pyogenic infection, a diagnosis of pancreatic enzyme-mediated inflammatory myositis, fasciitis, subcutaneous inflammatory changes suggestive of panniculitis, and sterile reactive knee synovitis associated with acute necrotizing pancreatitis was established.

The patient was managed conservatively with intravenous crystalloid resuscitation, analgesia, early enteral nutrition, correction of electrolyte abnormalities, and therapeutic anticoagulation for splanchnic venous thrombosis. Empirical antibiotics initiated during the initial evaluation were subsequently de-escalated in view of sterile cultures and the absence of imaging evidence of infection. Serial examinations demonstrated progressive reduction in limb swelling, improvement in knee range of motion, and complete resolution of fever without the need for surgical drainage, fasciotomy, or orthopedic intervention.

The patient was discharged on oral anticoagulation and supportive therapy. At 15-day clinical follow-up after discharge, he demonstrated marked improvement in lower limb pain and swelling, with significant recovery of knee movements and no recurrence of pancreatitis-related symptoms.

The patient had complete resolution of abdominal pain and was independently ambulant without residual functional deficit. As the patient demonstrated sustained clinical improvement, repeat imaging and additional laboratory investigations were not performed.

## Discussion

Musculoskeletal manifestations of acute pancreatitis are uncommon and frequently pose a diagnostic challenge because they may precede, accompany, or even overshadow abdominal symptoms. While pancreatic panniculitis and PPP syndrome are recognized extra-pancreatic manifestations, extensive inflammatory myositis with fascial involvement remains exceptionally rare. Our patient presented with severe acute necrotizing pancreatitis complicated by MRI-confirmed multicompartment inflammatory myositis, fascial inflammation, diffuse subcutaneous inflammatory changes suggestive of panniculitis, sterile reactive knee synovitis, and concomitant portal, splenic, and superior mesenteric venous thrombosis. To our knowledge, very few reports have documented this combination of findings with comprehensive clinicoradiological correlation.

The diagnosis of acute pancreatitis was established by characteristic abdominal pain, elevated serum lipase, and contrast-enhanced CT demonstrating pancreatic necrosis with multiple acute necrotic collections. The MCTSI of 8/10 indicated severe disease with a high inflammatory burden. Severe acute pancreatitis is characterized by marked activation of digestive enzymes and inflammatory mediators, resulting in endothelial dysfunction, increased vascular permeability, and systemic inflammatory response syndrome (SIRS), which together contribute to extra-pancreatic organ involvement [5,6].

The precise mechanism responsible for pancreatic enzyme-mediated musculoskeletal injury remains incompletely understood. The prevailing hypothesis suggests that lipase, phospholipase A₂, and other pancreatic enzymes escape into the systemic circulation following acinar cell injury. Lipase hydrolyzes triglycerides within adipocytes to generate free fatty acids, which combine with calcium to form insoluble soaps, producing enzymatic fat necrosis. This process triggers endothelial injury, activation of complement pathways, neutrophil recruitment, and release of pro-inflammatory cytokines including tumor necrosis factor-α, interleukin-1β, and interleukin-6. These inflammatory mediators further increase vascular permeability, allowing inflammatory edema to extend into the subcutaneous tissues, fascial planes, periarticular structures, and skeletal muscle. Histopathologically, pancreatic panniculitis is characterized by lobular fat necrosis with anucleate “ghost” adipocytes, although tissue confirmation was not obtained in our patient because there were no discrete nodular skin lesions amenable to biopsy and the patient demonstrated progressive clinical improvement with conservative treatment [7,8].

Unlike classical PPP syndrome, which is characterized by the triad of pancreatitis, panniculitis, and polyarthritis, our patient exhibited diffuse MRI-confirmed myositis with fascial inflammation and sterile reactive knee synovitis. Because panniculitis was diagnosed radiologically rather than histopathologically and inflammatory arthritis was limited to a single knee without biopsy confirmation, we believe the term “pancreatic enzyme-mediated musculoskeletal inflammation within the PPP syndrome spectrum” more accurately reflects the available evidence than a definitive diagnosis of classical PPP syndrome. This distinction is important because it acknowledges the biological continuum of pancreatic enzyme-mediated injury while avoiding overinterpretation of the clinical findings.

MRI proved crucial in establishing the diagnosis. MRI demonstrated diffuse T2-weighted hyperintensity and post-contrast enhancement involving the anterior, medial, and posterior muscle compartments together with extensive fascial fluid tracking and diffuse subcutaneous inflammatory changes. Importantly, there was no well-defined intramuscular abscess, osteomyelitis, cortical destruction, or marrow edema. MRI is the preferred imaging modality for inflammatory myopathies because of its excellent soft tissue contrast and its ability to differentiate diffuse inflammatory edema from pyogenic infection or muscle infarction [9].

The differential diagnosis in this patient was broad. Pyomyositis was initially considered because of fever, localized pain, and muscle swelling. However, pyomyositis typically progresses through invasive, suppurative, and late septic stages, with MRI demonstrating localized intramuscular abscesses and rim enhancement. Blood cultures may be positive in up to one-third of patients, and surgical drainage is frequently required. In contrast, our patient demonstrated diffuse multicompartment inflammatory edema without drainable collections, negative blood cultures, and progressive recovery without surgical intervention, making pyomyositis unlikely [10].

Necrotizing fasciitis was another important consideration because MRI demonstrated extensive fascial edema. However, necrotizing fasciitis usually presents with rapidly progressive pain, hemodynamic instability, cutaneous necrosis, hemorrhagic bullae, soft tissue gas, and extensive fascial necrosis requiring emergency surgical debridement. None of these features were present clinically or radiologically, and the patient’s condition improved without operative intervention [11].

Similarly, septic arthritis was considered because of painful knee swelling and joint effusion. Synovial fluid analysis, however, demonstrated only 250 leukocytes/mm³, substantially below the levels typically observed in septic arthritis. Furthermore, blood and urine cultures remained sterile, and MRI demonstrated no evidence of destructive arthritis or osteomyelitis. Collectively, these findings strongly favored sterile inflammatory synovitis rather than bacterial joint infection [12].

Deep venous thrombosis was excluded by duplex Doppler ultrasonography, which demonstrated normal venous compressibility and preserved arterial flow. This finding is clinically important because unilateral painful limb swelling in hospitalized patients is frequently attributed to venous thrombosis, potentially delaying recognition of inflammatory musculoskeletal complications associated with pancreatitis.

Other non-infectious causes of unilateral inflammatory myositis, including autoimmune inflammatory myopathies, diabetic muscle infarction, vasculitis, and drug-induced myopathy, were considered less likely because of the temporal association with acute pancreatitis, characteristic imaging findings, absence of suggestive clinical features, and recovery with treatment directed at pancreatitis.

Another notable feature of our case was the presence of portal vein, splenic vein, and superior mesenteric vein thrombosis. Splanchnic venous thrombosis occurs in approximately 15-20% of patients with necrotizing pancreatitis and results from local inflammation, endothelial injury, venous compression by pancreatic collections, and systemic hypercoagulability. Involvement of the superior mesenteric vein is particularly important because of the potential risk of intestinal ischemia. Therapeutic anticoagulation was initiated after careful assessment of bleeding risk and was associated with a favorable clinical outcome [13].

The management of pancreatic enzyme-mediated musculoskeletal inflammation differs fundamentally from that of infective soft tissue disorders. Although empirical antibiotics were appropriate during the initial evaluation because of fever and diagnostic uncertainty, definitive treatment focused on optimal management of acute pancreatitis, including intravenous fluid resuscitation, early enteral nutrition, adequate analgesia, correction of metabolic abnormalities, and anticoagulation for splanchnic venous thrombosis. Current international guidelines discourage prophylactic intervention for sterile pancreatic necrosis and recommend image-guided drainage or surgery only when infected necrosis or persistent clinical deterioration develops. Our patient’s progressive improvement without orthopedic surgery or fasciotomy emphasizes that recognition of this entity can prevent unnecessary invasive procedures [14].

This case has several strengths. It combines multimodality imaging, including CT, Doppler ultrasonography, musculoskeletal ultrasonography, and MRI, with microbiological evaluation and synovial fluid analysis, allowing systematic exclusion of important differential diagnoses. In addition, the coexistence of multicompartment myositis, fascial inflammation, reactive synovitis, and extensive splanchnic venous thrombosis illustrates the broad systemic inflammatory consequences of severe acute pancreatitis.

This report has several limitations. Histopathological confirmation of panniculitis was not obtained because there were no accessible lesions for biopsy and the patient improved with conservative management. Additional inflammatory markers, including erythrocyte sedimentation rate (ESR), C-reactive protein (CRP), procalcitonin, and creatine kinase, were not performed as part of routine clinical care.

## Conclusions

Acute necrotizing pancreatitis can rarely present with pancreatic enzyme-mediated musculoskeletal inflammation, manifesting as extensive unilateral lower limb myositis, fasciitis, and sterile reactive synovitis that closely mimics infectious or vascular disorders. This case highlights the importance of maintaining a high index of suspicion for extra-pancreatic inflammatory manifestations in patients with pancreatitis who develop unexplained painful limb swelling. Multimodality imaging, particularly MRI, together with exclusion of deep venous thrombosis and soft tissue infection, is essential for establishing the diagnosis and avoiding unnecessary surgical intervention. Early recognition and treatment directed at the underlying pancreatitis can result in favorable clinical outcomes and broaden awareness of this rare but important complication.
